# Macrophage migration inhibitory factor gene polymorphisms (SNP ‐173 G>C and STR‐794 CATT5‐8) confer risk of plaque psoriasis: A case–control study

**DOI:** 10.1002/jcla.23999

**Published:** 2021-09-17

**Authors:** Jorge Hernández‐Bello, Miroslaba Rodríguez‐Puente, Jorge Gutiérrez‐Cuevas, Samuel García‐Arellano, José Francisco Muñoz‐Valle, Mary Fafutis‐Morris, Delfina Guadalupe Villanueva‐Quintero, Anabell Alvarado‐Navarro

**Affiliations:** ^1^ Instituto de Investigación en Ciencias Biomédicas Centro Universitario de Ciencias de la Salud Universidad de Guadalajara Guadalajara Jalisco Mexico; ^2^ Dermika Centro Dermatológico y Láser. Vallarta Nte Guadalajara Jalisco Mexico; ^3^ Departamento de Biología Molecular y Genómica Instituto de Biología Molecular en Medicina y Terapia Génica Centro Universitario de Ciencias de la Salud Universidad de Guadalajara Guadalajara Jalisco Mexico; ^4^ Centro de Investigación en Inmunología y Dermatología Centro Universitario de Ciencias de la Salud Universidad de Guadalajara Guadalajara Jalisco México; ^5^ Centro de Atención en Enfermedades Inflamatorias Col. Lomas de Guevara Guadalajara Jalisco México

**Keywords:** genetic susceptibility, haplotypes, MIF, plaque psoriasis, polymorphisms

## Abstract

**Background:**

Macrophage inhibitory factor (MIF) is a pro‐inflammatory cytokine secreted by several cells, including those in the immune system and the skin. The *MIF* gene contains the SNP ‐173 G> C and STR ‐794 CATT_5‐8_ polymorphisms in the promoter region capable of affecting its activity. Our objective was to investigate the *MIF* polymorphisms as a risk factor for plaque psoriasis (PP) in the Mexican population.

**Methods:**

We genotyped both *MIF* polymorphism (rs5844572 and rs755622) in 224 PP patients with a clinical and histopathological diagnosis and 232 control subjects (CS) by the PCR‐RFLP method. MIF serum levels were determined by an ELISA kit.

**Results:**

We found significant differences in the genotypic and allelic frequencies for the *MIF* ‐173 G>C polymorphism; carriers of the GC genotype (OR 1.51, 95% CI 1.026–2.228, *p* = 0.03) and the C allele (OR 1.34, 95% CI 1.005–1.807, *p* = 0.04) had higher odds to present with PP. Moreover, the 6C haplotype was associated with PP risk (OR 2.10, 95% CI 1.22–3.69, *p *< 0.01). Also, the ‐173 CC genotype was associated with high MIF serum levels (*p *< 0.05).

**Conclusions:**

The ‐173 GC genotype and the 6C haplotype of the *MIF* polymorphisms are associated with susceptibility to PP in the Mexican population.

## Graphical abstract

Summary of key results from this study. (1) “Linkage disequilibrium was identified between ‐173 G>C and STR‐794 CATT5‐8 polymorphisms (D′ value = 0.62, r^2^ = 0.17, *p* < 0.001, Figure 1). (2) “the ‐173 CC genotype was associated with high MIF serum levels ". (3) "The ‐173 GC genotype and the 6C haplotype of the MIF polymorphisms are associated with susceptibility to PP in the Mexican population". (4) "overproduction of MIF may be important in mediating pathogenic effects in psoriasis".

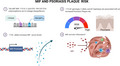

## 1 INTRODUCTION

Psoriasis is a highly prevalent skin disease that affects 2%–3% of the population worldwide.^1^ It is a chronic dermatosis characterized by well‐defined erythematous‐squamous plaques with excessive keratinocyte proliferation and bilateral symmetric distribution, located mainly on the elbows, knees, sacral region, and scalp.^2^, ^3^ The severity of psoriasis is determined according to the PASI index (Psoriasis Area Severity Index), which evaluates the extent, erythema, and thickness of the scales present in the affected area.^4^

The plaque‐type lesions are a consequence of the inflammation of the skin, epidermal hyperplasia, and angiogenesis due to dysregulation of the skin immune responses.^5^ Some authors have suggested that the cytokines expressed in psoriatic skin could explain most of the clinical features of this disease.^6^, ^7^

MIF is a cytokine synthesized by leukocytes and cells of tissues exposed to the environment, such as the epithelial lining of the skin.^8^ The skin and serum of psoriatic patients express increased levels of MIF, but its pathogenic function in psoriasis is still not clear.^9^

High MIF levels have been associated with two polymorphisms of the *MIF* gene (SNP ‐173 G>C and STR ‐794 CATT_5‐8_),^10^, ^11^ as well as with enhanced risk to develop autoimmune, infectious, and chronic inflammatory,^12^, ^13^, ^14^, ^15^ including psoriasis,^16^, ^17^ psoriatic arthritis, and systemic sclerosis in the Mexican population.^13^, ^18^, ^19^ However, its role as a marker of genetic susceptibility to psoriasis has been poorly explored.

This study aimed to determine the association between the SNP ‐173 G>C and STR ‐794 CATT_5‐8_ polymorphisms of the *MIF* gene with MIF serum levels and the risk of presenting plaque psoriasis in a Mexican mestizo population.

## 2 MATERIAL AND METHODS

### 2.1 Study subjects

This case and controls study included 224 patients with a clinical and histopathological diagnosis of plaque psoriasis. The sample size was calculated using the OpenEpi v. 3.01 software considering 23.6% as the frequency of the minor allele at ‐173 G>C polymorphism according to previous reports in the Mexican population.^20^ The patients were recruited from the Jalisco Dermatological Institute, "Dr. José Barba Rubio," of the Mexican Ministry of Health, Guadalajara, Jalisco, Mexico. In addition, 232 clinically healthy subjects were also included as controls, with no family relationship to the patients or a psoriasis history. The inclusion criteria for the subjects of both study groups were Mexican mestizo individuals over 18 years of age without any other chronic disease.

### 2.2 Ethical considerations

The research was carried out following the ethical aspects of the Declaration of Helsinki. Ethical approval (38/IDJ‐JAL/2017) was obtained from the Dermatological Institute of Jalisco, "Dr. José Barba Rubio." The participation of the subjects was voluntary, and their consent was formally written and signed by each individual.

### 2.3 Genomic DNA extraction

Genomic DNA (gDNA) was obtained from peripheral venous blood samples using Miller's modified salting‐out technique.^21^

### 2.4 Genotyping

The target sequence of the *MIF* gene was amplified by the polymerase chain reaction (PCR) technique, using the following conditions: an initial denaturation of 95°C for 4 min, followed by 30 cycles of denaturation at 95°C for 30 s, alignment at 60°C for 30 s, and extension at 72°C for 30 s; and a final extension at 72°C for 2 min. The products amplified by PCR were visualized in polyacrylamide gels (Sigma‐Aldrich) 29:1 and stained with 0.2% silver nitrate (Caledon, Canada).

The genotyping of SNP ‐173 G>C was performed by PCR, followed by restriction fragment length polymorphisms (PCR‐RFLPs) using *MIF* gene forward (5'‐ACTAAGAAAGACCCGAGG‐3') and reverse (5'‐GGGGCACGTTGGTGTTTACG‐3') primers (Sigma‐Aldrich), which generated a 366 base pair (bp) fragment.^22^ The final volume of the mixture was 15 µl, which was composed of 1× Buffer A, 0.12 U/µl *Taq* polymerase (Vivantis Technologies), 5 mM MgCl_2_, 0.1 mM dNTPs, 0.09 mM primers (Invitrogen), 1.2 mM betaine (Sigma Aldrich), and gDNA 100 ng/µl as substrate.

**FIGURE 1:**
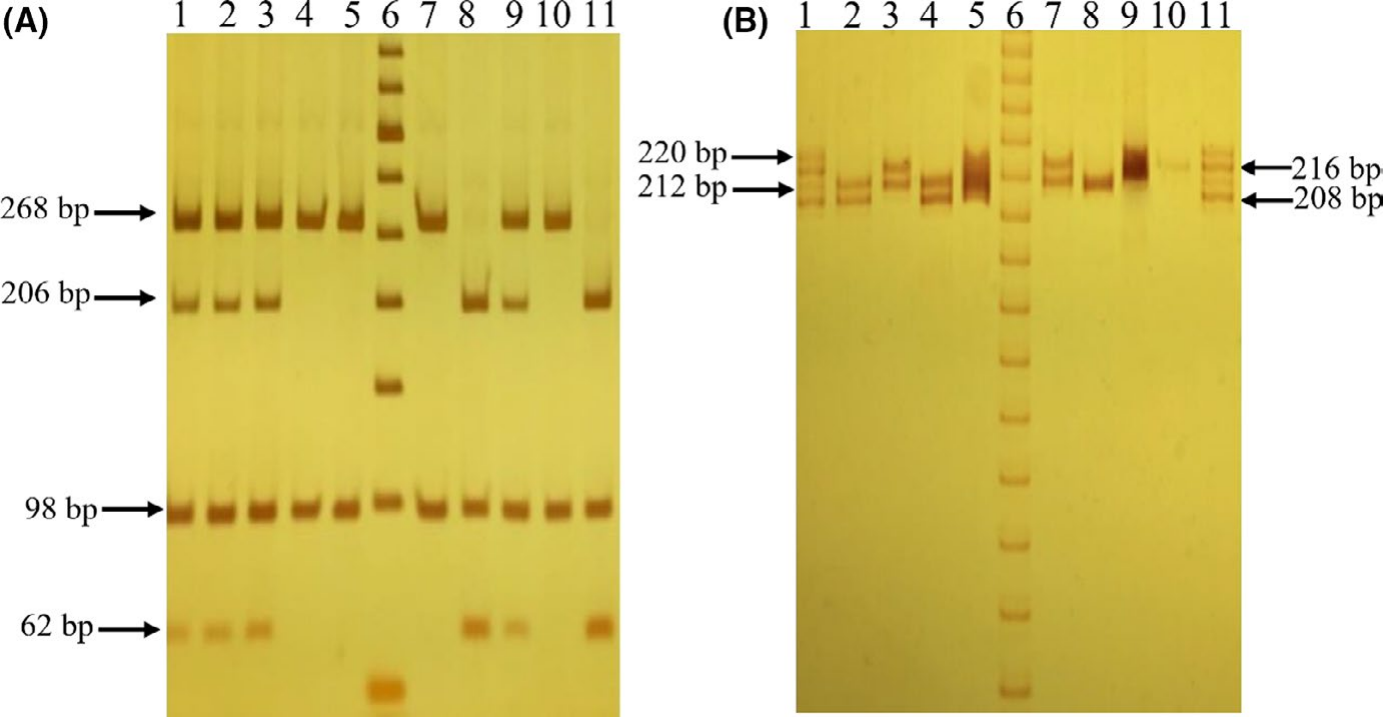
Genotypes identification of the SNP ‐173 G>C and STR‐794 CATT5‐8 of the *MIF* gene. The figures show polyacrylamide gels. (A) SNP ‐173 G>C: lanes 4,5,7,10 (GG genotypes); lanes 1–3,9 (GC genotypes); lanes 8,11 (CC genotypes); lane 6 (molecular weight marker of 50 bp). (B) STR‐794 CATT_5‐8_: lanes 2,4 (5,6 genotypes); lane 3,7 (6,7 genotypes); lanes 5,9 (undetermined genotypes); lane 8 (6,6 genotype); lane 10 (7,7 genotype); lanes 1, 11 (allelic ladder of the four repetitions); lane 6 (molecular weight marker of 10 bp). Undetermined samples were analyzed in duplicate on a new electrophoresis

The amplified products were digested with the restriction enzyme *AluI* (New England BioLabs) for 16 h at 37°C. The enzyme generated restriction fragments according to the genotype; in all the amplified products, an additional fragment was obtained 98 bp, due to the presence of another restriction site: CC wild‐type homozygous generates three bands (206, 98, and 62 bp); heterozygous GC generates four bands (268, 206, 98, and 62 bp) and homozygous polymorphic GG generates two bands (268 and 98 bp). The obtained products were visualized on 6% polyacrylamide gels (Figure 1A). The results were confirmed by sequencing some randomly selected samples.

The STR ‐794 CATT_5‐8_ polymorphism was genotyped as reported by a previously described protocol.^18^ Fragments were generated according to genotype: Allele 5 generated a 208 bp band, allele 6 a 212 bp, 7 a 216 bp, and 8 a 220 bp band. The PCR amplified products were visualized by electrophoresis on 10% polyacrylamide gels (Figure 1B).

### 2.5 MIF serum levels

MIF serum levels were quantified using the commercial ELISA kit, Human MIF Immunoassay (R & R&D). The procedure was carried out following the fabricant's instructions. The sensitivity of the assay was 6 pg/ml.

### 2.6 Statistical analysis

The determination of the genotypic and allelic frequencies of the polymorphisms was carried out by direct counting. The Hardy–Weinberg equilibrium was calculated in the control group. Genotypic and allelic frequencies of the SNP ‐173 G>C and STR‐794 CATT_5‐8_ polymorphisms in patients and CS were analyzed by Chi‐square using 2 × 2 contingency tables. The association analysis between genotypes and psoriasis was estimated by odds ratio (OR) with a 95% confidence interval, considering a significance level of *p* < 0.05. Linkage disequilibrium was evaluated with the SHEsis software. The statistical analyses were done with the Stata 9.0 software and GraphPad Prism version 8.0.2.

## 3 RESULTS

### 3.1 Clinical characteristics of study subjects

Clinical and demographic characteristics of PP patients and CS are described in Table 1. PP patients had a median age of 50 (36–59) years, and 62% were male. The average time of evolution of the disease was 10 years, and the median of patients had a PASI score of 6.

**TABLE 1:** Clinical and demographic characteristics of PP patients and CS

Characteristics	PP (*n* = 224)	CS (*n* = 232)	*p* Value
Age^‡^, ^†^	50 (36–59)	38 (25–48)	<0.0001
Gender, *n* (%)			
Female	86 (38)	129 (56)	<0.001
Male	138 (62)	103 (44)	
Time of evolution of the disease^§^, ^¶^	10 ± 0.63	‐	
PASI^§^, ^¶^	6 ± 0.46	‐	
Treatment, *n* (%)			
Topical (exfoliants)	78 (35)	‐	
Systemic	77 (34)	‐	
Without	69 (31)	‐	
Comorbidities, *n* (%)			
Obesity	15 (7)	‐	
Hypertension	25 (11)	‐	
Hypercholesterolemia	9 (4)	‐	

Abbreviations: CS, Control subjects; PP, plaque psoriasis patients.

^‡^ Data are expressed as median (p25th–p75th).

^§^ Data are expressed as mean ± standard error.

Some of the PP patients were using treatments when the study started. Out of them, 35% were treated with topical exfoliants, 34% with systemic treatment, and 31% were without treatment. The median age of the CS was 38 (25–48) years, and most of them were female (56%); both characteristics were different compared to PP patients (*p *< 0.001).

### 3.2 Frequencies of the ‐794 CATT_5–8_ and ‐173 G>C of *MIF* polymorphisms and their haplotypes

The distribution of the alleles, genotypes, and haplotypes of *MIF* polymorphisms (‐794 CATT_5‐8_ and ‐173 G>C) in PP patients and CS is shown in Table 2. The 6,6 genotype of the ‐794 CATT5‐8 polymorphism was the most frequent in PP patients (31%) and CS (33%). On the other hand, the GG genotype of the ‐173G>C polymorphism was the most frequent in both study groups (PP 48% vs. CS 58%). Both polymorphisms were in Hardy–Weinberg equilibrium in the CS group (*p *> 0.05). We found significant differences in the genotypic and allelic frequencies only for the *MIF* ‐173 G>C polymorphism: GC genotype (OR 1.51, 95% CI 1.026–2.228, *p* = 0.03) and C allele (OR 1.34, 95% CI 1.005–1.807, *p* = 0.04) were at higher odds to be present in PP patients. This same association was observed with a dominant inheritance model (OR 1.51, 95% CI 1.043–2.187, *p* = 0.02).

**TABLE 2:** Genotype and allele frequencies of ‐794 CATT_5‐8_ and ‐173 G>C *MIF* polymorphisms in PP patients and CS

Polymorphism	PP *n *= 224% (*n*)	CS *n *= 232% (*n*)	OR (CI 95%)	*p* Value‡, †
MIF ‐794 CATT_5‐8_ (rs5844572)				
Genotype				
5,5	3.5 (8)	3 (6)	1.47 (0.421–5.403)	0.49
5,6	25 (56)	29 (68)	0.91 (0.544–1.510)	0.69
5,7	9.5 (22)	5 (12)	2.02 (0.880–4.822)	0.07
5,8	0.5 (1)	‐	‐	‐
6,6^§^, ^¶^	31 (69)	33 (76)	1	‐
6,7	23 (51)	23 (54)	1.04 (0.615–1.770)	0.88
6,8	0.5 (1)	1 (2)	0.55 (0.010–10.830)	0.62
7,7	7 (16)	6 (14)	1.26 (0.534–3.012)	0.57
Allele				
5	21 (95)	20 (92)	1.16 (0.820–1.640)	0.39
6^§^, ^¶^	55 (246)	59.5 (276)	1	‐
7	23.5 (105)	20 (94)	1.25 (0.891–1.763)	0.17
8	0.5 (2)	0.5 (2)	‐	**‐**
MIF ‐173 G>C (rs755622)				
Genotype				
GG^§^, ^¶^	48 (107)	58 (135)	1	‐
GC	44 (99)	35 (82)	1.51 (1.026–2.228)	**0.03**
CC	8 (18)	7 (15)	1.51 (0.724–3.121)	0.26
Allele				
G^§^, ^¶^	70 (313)	76 (352)	1	‐
C	30 (135)	24 (112)	1.34 (1.005–1.807)	**0.04**
Genetic models				
*Dominant*				
GG^§^, ^¶^	48 (107)	58 (135)	1	‐
GC+CC	52 (117)	42 (97)	1.51 (1.043–2.187)	**0.02**
*Recessive*				
GG+GC^§^, ^¶^	92 (206)	93 (217)	1	
CC	8 (18)	7 (15)	1.25 (0.618–2.563)	0.52

Abbreviations: PP, plaque psoriasis patients; CS, control subjects; OR, odds ratio; CI, confidence interval.

^†^ Chi‐square test.

^¶^ Reference category.

Bold values denote statistical significance at the *p* < 0.05 level.

**FIGURE 2:**
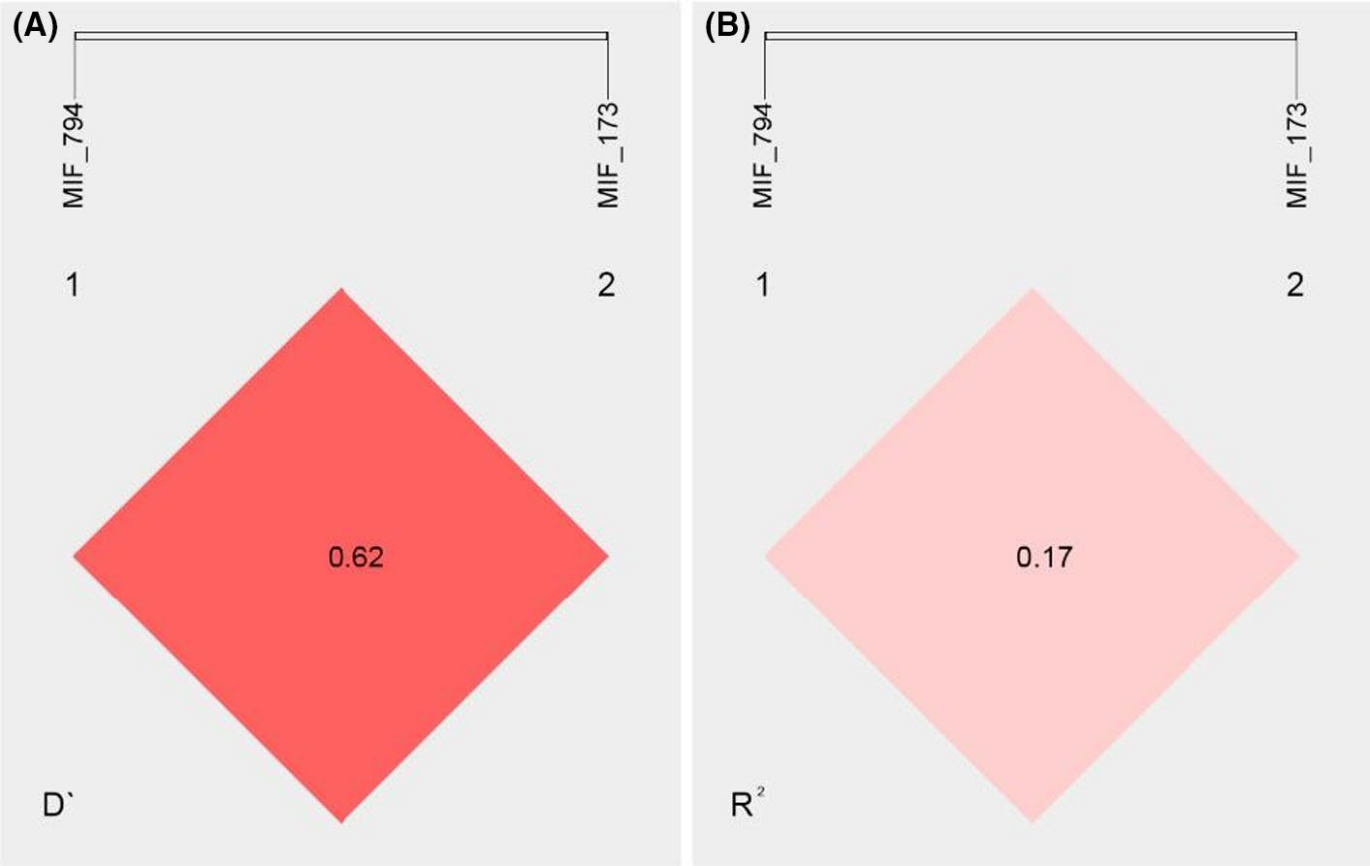
Pairwise linkage disequilibrium relationships between the *MIF* ‐794 CATT_5–8_ and *MIF* ‐173 G>C variants. (A) Lewontin's coefficient *D*′ and (B) the correlation coefficient *R* ^2^ was calculated using SHEsis software

Linkage disequilibrium was identified between both *MIF* polymorphisms (D′ value = 0.62, r^2^ = 0.17, *p* < 0.001, Figure 2). The 6G was the most frequent haplotype in the PP patients and the CS (45% and 54%, respectively). The 6C haplotype was at higher odds to PP (OR 2.10, 95% CI 1.22–3.69, *p *< 0.01) (Table 3). The demographic and clinical characteristics were not associated with these genotypes or *MIF* haplotypes (data not shown).

**TABLE 3:** Haplotype frequencies of ‐794 CATT_5‐8_ and ‐173 G>C *MIF* polymorphisms in PP and CS

Haplotype	PP % (*n* = 448)	CS % (*n* = 464)	OR (CI 95%)	*p* Value
5C	1 (5)	2 (8)	0.78 (0.20–2.74)	0.66
5G	20 (90)	18 (84)	1.33 (0.92–1.92)	0.11
6C	10 (44)	6 (26)	2.10 (1.22–3.69)	**<0.01**
6G^¶^	45 (201)	54 (250)	1	‐
7C	19 (84)	16 (77)	1.36 (0.93–1.98)	0.10
7G	5 (21)	4 (17)	1.54(0.75–3.19)	0.20
8C	0 (2)	0 (1)	2.47 (0.13–147)	0.44
8G	0 (1)	‐	‐	**‐**

Abbreviations: PP, Plaque psoriasis patients; CS, control subjects; OR, odds ratio; CI, confidence interval.

^¶^ Reference category. The *p*‐value was calculated by logistic regression comparison with the reference haplotype 6G.

### 3.3 *MIF* serum levels in PP patients and CS

**FIGURE 3:**
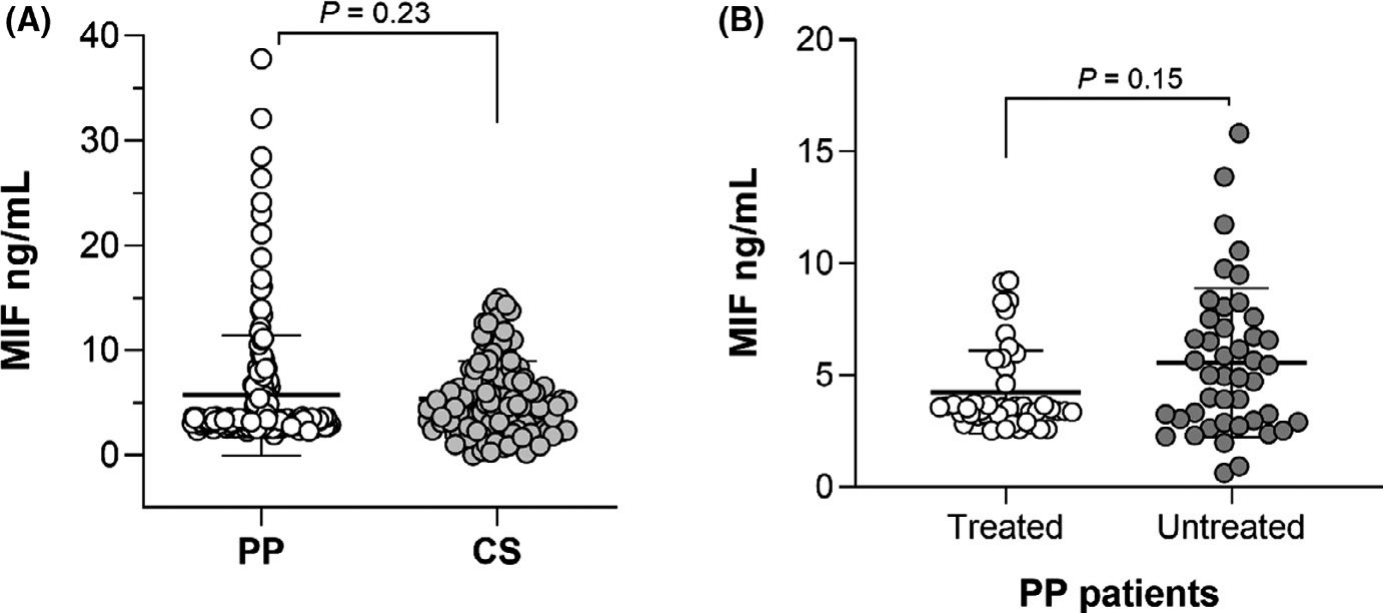
Comparison of MIF serum levels. (A) MIF serum levels of PP patients and CS. (B) Comparison of MIF serum levels by treatment in PP patients. *p*‐Value was calculated using U‐Mann–Whitney; the data are provided in medians and p25th–p75th

We did not find significant differences of MIF serum levels between PP patients and CS (Figure 3A, *p* = 0.23). In addition, MIF serum levels were not related to the treatment in the PP patients’ group (Figure 3B, *p* = 0.15).

**FIGURE 4:**
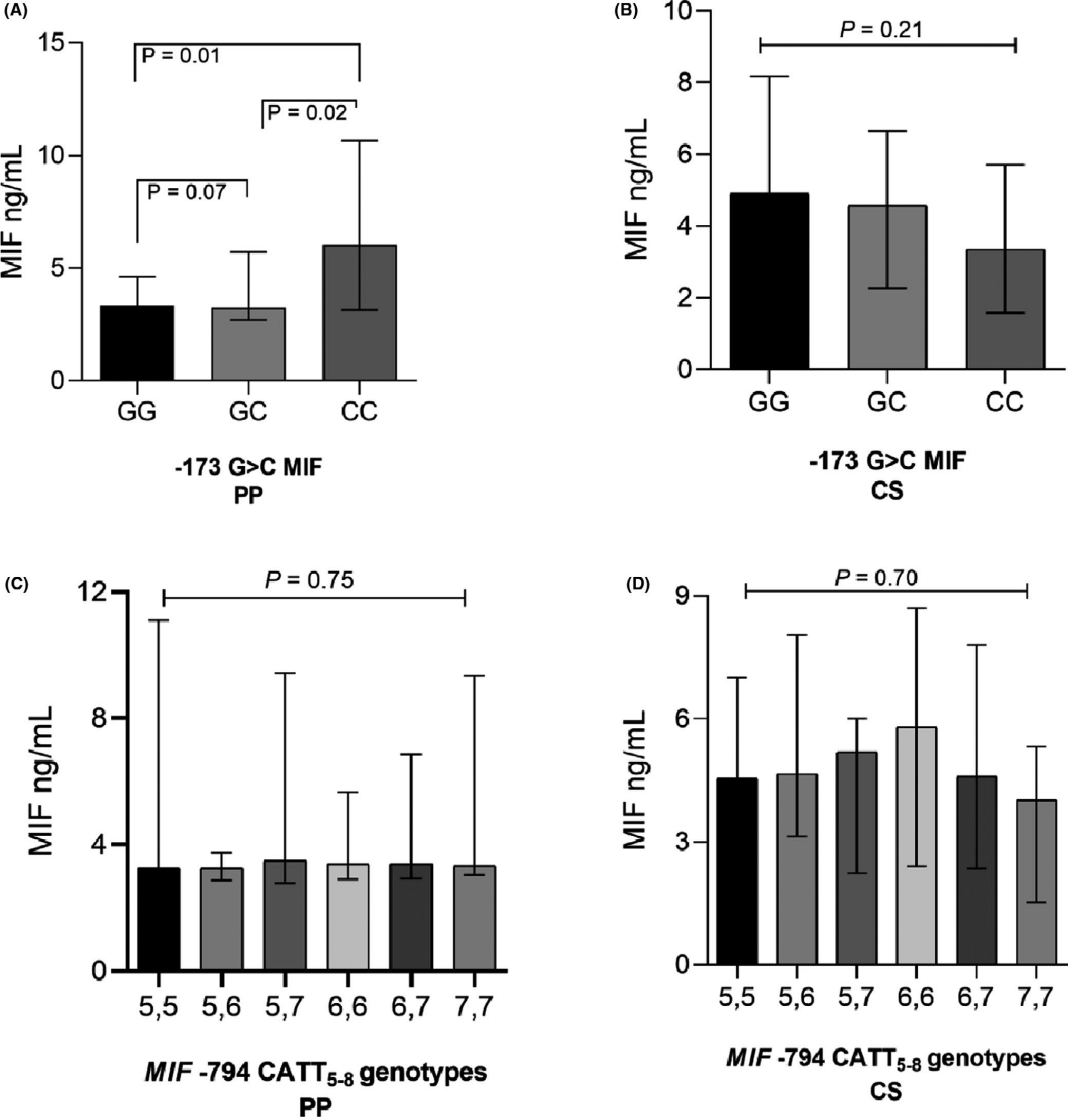
MIF serum levels according to ‐173 G>C and ‐794 CATT_5‐8_ *MIF* polymorphisms in PP patients and CS. MIF serum levels according to *MIF* ‐173 G > C genotypes in plaque psoriasis (PP) patients (A) and CS (B). C and D show MIF serum levels according to different *MIF* ‐794 CATT_5‐8_ genotypes in PP patients and CS, respectively. *p*‐Values were calculated by the Kruskal–Wallis test, followed by Dunn's adjustment for multiple comparisons. The data are provided in medians and p25th–p75th. PP, Plaque psoriasis patients; CS, control subjects

Also, the MIF serum levels were not associated with the clinical variables of the PP patients (data not shown). However, the ‐173 G>C polymorphism genotypes were associated with differences in MIF serum levels in PP patients (Figure 4A). In general, the CC genotype carriers had higher MIF serum levels than those carrying the GC (*p* = 0.02) or GG genotypes (*p* = 0.01).

On the other hand, the *MIF* ‐794 CATT5–8 STR genotypes did not show an association with MIF levels in the study groups (Figure 4C,D). MIF serum levels were also analyzed according to *MIF* haplotypes, but we did not find significant differences (data not shown).

## 4 DISCUSSION

The main pathological manifestations of psoriasis are inflammation, keratinocyte hyper‐proliferation, altered maturation of the epidermis, and vascular alterations. Therefore, a combination of immunologic disarrangement, psoriasis‐associated susceptibility loci, autoantigens, and multiple environmental factors could be the cause of this multifactorial disease.^23^

Genome‐wide association studies in psoriasis patients have identified that polymorphisms within or near some genes encoding cytokines are implicated in the pathomechanism of this disease.^6^ A previous study reported by Shimizus et al. showed that MIF participates in the pathogenesis of PP by the elevated serum levels of this cytokine in patients and the spontaneous release of higher amounts of MIF derived from PBMCs of patients compared to CS.^24^ However, we did not find statistical differences in the serum levels of MIF between PP patients and CS, even when comparing the MIF levels between patients under treatment and those untreated.

A possible explanation for these discrepancies is that Shimizus et al. determined MIF serum levels in untreated patients with active psoriasis (PASI score ≥20) in contrast with us, as 71% of our PP patients were under pharmacological treatment and with mild psoriasis (PASI = 6 ± 0.46). Although we did not find statistical differences in MIF levels by treatment, a trend of higher MIF levels is evident in those patients without treatment (median of MIF serum levels: 3.5 ng/ml vs. 4.9 ng/ml, respectively). On the other hand, if we assume that MIF has a key role in the severity of psoriasis, the lack of correlation in our study could be explained by the low PASI score of our patients.

It could be argued that MIF may play a role in the evolution of psoriatic lesions but not a major role at the systemic levels. This hypothesis is supported by Steinhoff et al., who only reported increased MIF expression in endothelial cells of psoriatic lesions.^25^ Conversely, Shimizu et al. reported a reduced expression of MIF expression in psoriatic skin lesions. They interpreted this reduction as a counter‐regulation in response to high serum levels and that these discrepancies could be explained by the clinical stages of patients, the exact area of examined skin lesions, and whether these were exposed to sunlight.^24^

To explain the aforementioned discrepancies, more studies in tissue lesions are needed to clarify the exact role of MIF in the pathogenesis of psoriasis, mainly studies controlling clinical variables such as the disease activity, stage of the disease, ethnicity, or treatment. All of these would support the suggestions of MIF as a target for future therapies in psoriasis or other skin diseases.

In inflammatory skin diseases, as systemic sclerosis, the fibroblast and mononuclear infiltrating cells produce MIF in skin tissue. This action could be performed through an autocrine/paracrine mechanism, suggesting that MIF participates in the local pro‐inflammatory loop that leads to tissue remodeling.^26^

Genetic studies have uncovered several loci associated with susceptibility to psoriasis, highlighting the pathogenic involvement of genes related to inflammation; however, not all the underlying genes have been conclusively identified.^27^ We observed a significant association between the *MIF* ‐173 C allele and the *MIF* ‐173 GC genotype with PP risk. This association remained consistent with the dominant genetic inheritance model, which suggests that the presence of a single C allele is sufficient to confer risk of this disease. These results were similar to those observed in UK Caucasian patients with plaque psoriasis.^17^

The minor *MIF* ‐173 C allele or the GC genotype has also been associated with other diseases such as rheumatoid arthritis, juvenile idiopathic arthritis, systemic sclerosis, systemic lupus erythematosus, vitiligo, and sarcoidosis.^12^, ^19^, ^28^, ^29^, ^30^, ^31^ This association indicates that the *MIF* gene could be a common genetic marker of the autoimmune and inflammatory diseases of the skin.

The link between *MIF* ‐173 C allele and the disease's risk remains unclear, as most studies focus on genotyping data and do not evaluate circulating MIF levels or *MIF* mRNA expression to establish the functional effects of this genetic variant. Nevertheless, we studied circulating levels of MIF and found a relationship between this allele and higher serum levels of MIF in patients with psoriasis. This finding agrees with previous observations^17^, ^32^, ^33^, ^34^ and supports the functional effect of this polymorphism, which has been related to a binding site for protein activator‐4 (AP4) that increases promoter activity of *MIF*.^35^

The *MIF* CATT_5‐8_ STR was not associated with PP risk; however, the 6C haplotype (CATT_6_–*MIF* ‐173 C) was associated with an increased risk of susceptibility to PP. This association could be explained because the 6C haplotype has been associated with increased expression of *MIF* mRNA compared to those individuals carrying the 5G and 7C haplotypes.^36^ This is in agreement with a study in UK patients with psoriasis^17^ and with those reporting an association of this haplotype with increased susceptibility to juvenile idiopathic arthritis^29^ and adult inflammatory polyarthritis.^37^

To the best of our knowledge, this is the first study of the association between *MIF* polymorphisms and the susceptibility of PP in the Mexican population. Studies involving ethnically diverse populations and more detailed clinical manifestations should be conducted to verify our findings.

Some limitations of our study should be considered, such as the heterogeneity of comorbidities and treatments of our study subjects, which could influence the MIF levels. Moreover, the relation between the SNPs, MIF expression, and serum levels should be analyzed to further explore the effects of SNPs on serum levels and hence as a risk for PP.

In conclusion, we have provided evidence that the *MIF* ‐173C and *MIF* 6C haplotype (CATT_6_/‐173C) are associated with PP disease risk. These data also support the association of the *MIF* ‐173C allele with higher MIF serum levels, suggesting that overproduction of MIF may be important in mediating pathogenic effects in psoriasis.

## CONFLICT OF INTEREST

There is no conflict of interest in this work.

## ETHICAL APPROVAL

All experiments involving human subjects were conducted as per the ethical standards of the institutional and/or national research committee as well as the 1964 Helsinki Declaration and its later amendments or comparable ethical standards.

## INFORMED CONSENT

Informed consent was obtained from all individuals.

## CONSENT FOR PUBLICATION

All the contributing authors agreed for the publication in this journal.

## Data Availability

All the data related to this work are avsailable at the corresponding author. The data used to support the findings of this study are included in the article.
